# Lasting immune memory against hepatitis B in 12–13-year-old adolescents previously vaccinated with 4 doses of hexavalent DTPa-HBV-IPV/Hib vaccine in infancy

**DOI:** 10.1080/21645515.2016.1202388

**Published:** 2016-09-21

**Authors:** Ulrich Behre, Olivier Van Der Meeren, Priya Crasta, Linda Hanssens, Narcisa Mesaros

**Affiliations:** a Pediatric Practice, Kehl, Baden-Württemberg, Germany; b GSK Vaccines, Wavre, Belgium; c GSK Vaccines, Bangalore, India

**Keywords:** Adolescents, anamnestic response, challenge dose, DTPa-HBV-IPV/Hib, hepatitis B, immune memory, long-term, persistence, seroprotection

## Abstract

**Background:** Vaccinating infants against hepatitis B virus (HBV) is the most effective way of preventing the disease. However, since HBV exposure can increase during adolescence, it is essential that antibody persistence is maintained. We evaluated the antibody persistence and immune memory against hepatitis B, in 12-13 y olds who had received complete primary + booster vaccination with diphtheria-tetanus-acellular pertussis-hepatitis B-inactivated poliovirus/*Haemophilus influenza* type b (DTPa-HBV-IPV/Hib) vaccine in infancy.

**Methods:** Open phase-IV study conducted at 12 centers in Germany [NCT02052661]. Adolescents aged 12-13 y, vaccinated with 4 doses of DTPa-HBV-IPV/Hib (*Infanrix hexa*™, GSK Vaccines) in infancy, received a single challenge dose of monovalent pediatric hepatitis B vaccine (*Engerix*™-B Kinder; GSK Vaccines). Blood samples were taken before and 1-month post-challenge to measure anti-hepatitis B (anti-HBs) antibodies using a chemiluminescence immunoassay (seroprotection cut-off: ≥10 mIU/ml). Post-challenge adverse events (AEs) were monitored.

**Results:** 300 subjects were vaccinated; of 293 subjects in the ATP immunogenicity cohort, 60.5% had pre-challenge anti-HBs antibodies ≥10 mIU/ml, which rose to 97.6% post-challenge (≥100 mIU/ml in 94.1%). An anamnestic response was seen in 96.5% subjects. A 150-fold increase in antibody geometric mean concentrations was observed (22.4 to 3502.6 mIU/ml). Pain (44%) and fatigue (24.3%) were the most frequent solicited local and general AEs, respectively; 14.7% subjects reported unsolicited symptoms during the 31-day post-vaccination period. Two vaccine-unrelated serious AEs occurred.

**Conclusion:** Vaccination with DTPa-HBV-IPV/Hib in infancy induces sustained seroprotection and immune memory against HBV, as shown by the strong anamnestic response to the hepatitis B vaccine challenge in 12-13 year-old adolescents.

## Introduction

Hepatitis B virus (HBV) is a major global public health problem. Indeed, more than 250 million of 2 billion HBV infected individuals are chronic carriers.^1,2^ Vaccination against HBV in early childhood is the most effective way of preventing the infection.^3^ This strategy has significantly reduced the incidence and prevalence of HBV and associated complications.^4^ Studies conducted after primary immunization with hepatitis B vaccine in different populations have consistently shown long-term persistence and immune memory against HBV, lasting for up to 20 y.^5-7^

Combination vaccines are widely used to reduce the need for multiple injections and improve compliance, especially during the crowded infant vaccination schedules. Several combined vaccines containing hepatitis B vaccine are commercially available, including the hexavalent diphtheria-tetanus-pertussis-hepatitis B-inactivated poliomyelitis and *Haemophilus influenzae* type b conjugate vaccine (DTPa-HBV-IPV/Hib; *Infanrix hexa*™; GSK Vaccines). The immunogenicity of primary vaccination with DTPa-HBV-IPV/Hib vaccine has been well established in clinical trials and post-marketing studies,^8,9^ and further, long-term immunogenicity against HBV for at least 10 y has been demonstrated.^4,5^ However, the exact duration of protection provided by the DTPa-HBV-IPV/Hib vaccine against HBV, and the subsequent requirement for booster vaccination against HBV, to sustain immunity, is not known.

DTPa-HBV-IPV/Hib vaccination was introduced into the German routine immunization program in 2000;^10^ primary vaccination of infants involves 3 vaccine doses at 2, 3 and 4 months of age, followed by a booster dose at 11–14 months.^11^ In order to assess long-term antibody persistence and immune memory against HBV, we set up a series of 4 studies with increasing follow-up times of children who received 4 doses of DTPa-HBV-IPV/Hib in routine clinical practice. The previous 2 studies assessed antibody persistence in children at 4–5^4^ and 7–8^12^ y of age and this third study evaluated adolescents aged 12–13 y.

## Results

### Demographics

**Figure 1.**
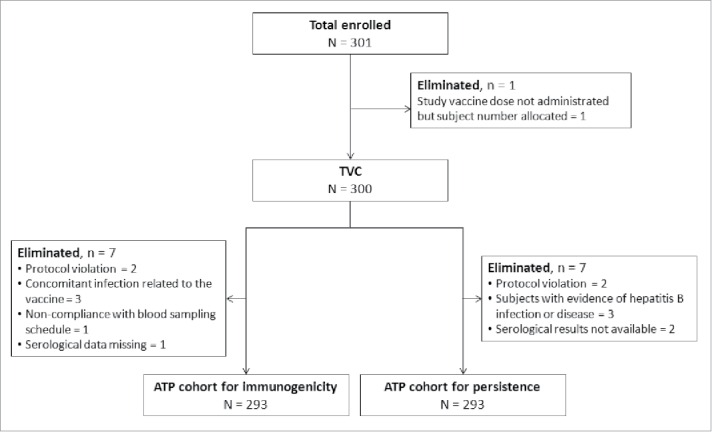
Subject disposition. ATP: According to protocol; TVC: Total vaccinated cohort.

Of 301 enrolled subjects, 300 were vaccinated and constituted the total vaccinated cohort (TVC) cohort. Seven subjects were excluded from the according-to-protocol (ATP) cohorts for immunogenicity and persistence (Fig. 1), including 3 who were eliminated because of ‘concomitant infection related to the vaccine’ and ‘evidence of hepatitis B disease’, respectively. It should be noted that none of these subjects had a medical history of hepatitis (i.e. were eligible for inclusion in the study) or recorded any signs or symptoms of hepatitis during the study. These 3 subjects were eliminated from the analyses after providing hepatitis B core antigen (HBc) positive results at the post-challenge time-point.

The mean age of the 293 subjects in the ATP cohort for immunogenicity was 12.3 y (standard deviation [SD]: 0.5 y); 50.5% subjects were male and the majority (99%) were Caucasian/European.

### Post-challenge immunogenicity

One month after the challenge dose, 97.6% (95% confidence interval [CI]: 95.1–99.0) of subjects were seroprotected and 94.1% (95% CI: 90.7–96.5) had anti-HBs levels ≥100 mIU/mL. The corresponding anti-HBs geometric mean concentration (GMC) was 3502.6 mIU/mL (95% CI: 2672.0–4591.5), representing an increase of over 150-fold compared with the pre-challenge anti-HBs GMC (Table 1).

**Table 1.** Antibody persistence (ATP cohort for persistence) and response to challenge (ATP cohort for immunogenicity) stratified according to the pre-challenge dose status.

anti-HBs antibody concentrations	Timing	N	% ≥ 6.2 mIU/mL (95% CI)	% ≥ 10 mIU/mL (95% CI)	% ≥ 100 mIU/mL (95% CI)	GMC mIU/mL (95% CI)
<6.2mIU/mL	Pre	88	-	-	-	3.1 (3.1–3.1)
	Post	89	93.3 (85.9–97.5)	92.1 (84.5–96.8)	82 (72.5–89.4)	476.1 (300.0–755.6)
≥6.2–<10mIU/mL	Pre	27	100 (87.2–100)	-	-	7.7 (7.1–8.3)
	Post	26	100 (86.8–100)	100 (86.8–100)	96.2 (80.4–99.9)	1739.8 (944.8–3203.8)
≥10mIU/mL	Pre	176	100 (97.9–100)	100 (97.9–100)	34.7 (27.7–42.2)	70.9 (58.0–86.7)
	Post	174	100 (97.9–100)	100 (97.9–100)	100 (97.9–100)	10792.2 (8354.3–13941.6)
Overall	Pre	291	69.8 (64.1–75.0)	60.5 (54.6–66.1)	21.0 (16.4–26.1)	22.4 (18.2–27.5)
	Post	289	97.9 (95.5–99.2)	97.6 (95.1–99.0)	94.1 (90.7–96.5)	3502.6 (2672.0–4591.5)

N: number of subjects with available results; %: percentage of subjects with concentration within the specified range; 95% CI: 95% confidence interval; GMC: geometric mean concentration calculated on all subjects; Pre: pre challenge dose time-point; Post: post challenge dose time-point.

Of all initially seronegative children, 93.3% were seropositive one month after the challenge dose, while 92.1% were seroprotected (anti-HBs antibody levels ≥10 mIU/mL). In these initially seronegative children, the lower limit of the 95% CI for the post-challenge GMCs was 300 mIU/mL, which is far above the seroprotective threshold (10 mIU/mL).

An anamnestic response was observed in 96.5% (277/287; 95% CI: 93.7–98.3) of subjects (Table 2). When stratified by pre-challenge serostatus, anamnestic responses to the HBV vaccine challenges were mounted by 92.0%, 100% and 98.3% of subjects with pre-challenge anti-HBs antibody levels <6.2 mIU/mL, ≥6.2–<10 mIU/mL and ≥100 mIU/mL, respectively.

**Table 2.** Anamnestic response to the hepatitis B vaccine challenge dose based on pre-challenge serostatus (ATP cohort for immunogenicity).

Pre-vaccination status	N	Anamnestic response % (95% CI)
<6.2mIU/mL	87	92.0 (84.1–96.7)
≥6.2–<10mIU/mL	26	100 (86.8–100)
≥10mIU/mL	174	98.3 (95.0–99.6)
Overall	287	96.5 (93.7–98.3)

N: number of subjects with both pre- and post-vaccination results available**;** %: percentage of responders; 95% CI: 95% confidence interval.

**Figure 2.**
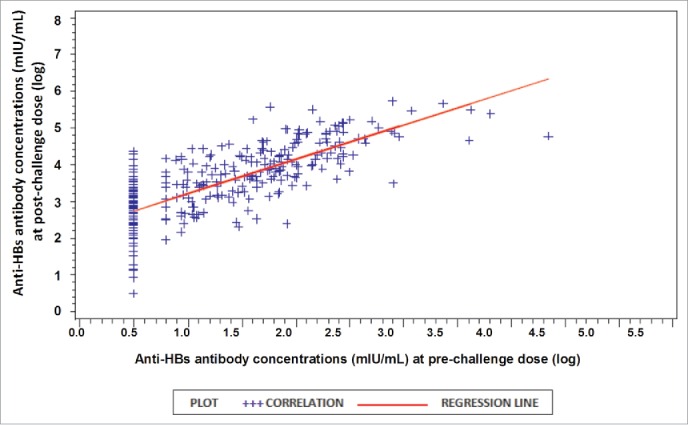
Anti-HBs antibody concentrations post-challenge as a function of pre-challenge concentrations, with regression line (ATP cohort for immunogenicity). Regression equation and R^2^ is given by: y = 2.2682+0.9386(x); R^2^ = 0.5254. Where, y = post challenge dose (log); x = pre challenge dose (log); R^2^ = proportion of variation in post challenge dose (log) that is predictable from pre challenge dose (log).

As shown in Figure 2, the magnitude of the post-challenge antibody response was related to persisting anti-HBs antibodies. About 53% of the variation in the response variable (post-challenge dose results) is explained by the pre-challenge dose results (R^2^ **=** 0.5254).

### Pre-challenge persistence

Before the challenge dose, 70% of subjects (95% CI: 64.4–75.2) in the ATP cohort for persistence remained seropositive for anti-HBs antibodies (anti-HBs levels ≥6.2 mIU/mL) and 60.8% (95% CI: 54.9–66.4) were seroprotected (anti-HBs levels ≥10 mIU/mL). The mean GMC was 22.7 mIU/mL (95% CI: 18.5–27.9).

In the ATP immunogenicity cohort, 60.5% of subjects had pre-challenge anti-HBs antibodies ≥10 mIU/mL.

### Reactogenicity and safety of the challenge dose

During the 4-day post-challenge follow-up period, at least one solicited or unsolicited local/general symptom was reported for 67.0% (95% CI: 61.4–72.3) of subjects. Pain at the injection site (44%; 95% CI: 38.3–49.8) and fatigue (24.3%; 95% CI: 19.6–29.6) were the most frequent solicited local and general symptoms, respectively; injection site swelling (0.7%) and headache (2.3%) were the most frequent local/general Grade 3 symptoms, respectively.

At least one unsolicited adverse event (AE) was reported in 44 (14.7%; 95% CI: 10.9–19.2) subjects during the 31-day post-vaccination follow up period. Upper respiratory tract infection was the most frequent unsolicited AE observed in 10 subjects. Five subjects had at least one grade 3 unsolicited AE (abdominal pain, pyrexia, gastrointestinal infection, contusion, headache and cough). Two subjects had at least one unsolicited AE, considered by the investigator to be vaccine-related (vertigo and urticaria).

Two subjects recorded serious adverse events (SAEs; contusion and forearm fracture), neither of which was considered by the investigator to be causally related to the vaccination. No subject withdrew from the study due to any AE/SAE.

## Discussion

Primary immunisation with hepatitis B vaccine in different populations have consistently shown long-term persistence and immune memory against HBV, lasting for up to 20 y, and various studies assessed long-term antibody persistence and responses to challenge doses 4 to 13 y after a primary vaccination.^4,6,10,12,13^ This current study is one of a 4 study series assessing long term antibody persistence and immune memory in increasingly older age groups. The first 2 studies evaluated 4–5 and 7–8 year-old children^4,12^ and this study was performed in 12–13 year-old German adolescents vaccinated with 4 doses of DTPa-HBV-IPV/Hib in infancy as part of routine clinical practice. In this study, the challenge dose was administered to adolescents to mimic the immunological response upon re-exposure to hepatitis B virus, and therefore served as an indicator of immune memory.

The main finding from this study is that DTPa-HBV-IPV/Hib vaccine given in infancy provides good protection against HBV infection for at least 12–13 y, with 60.8% subjects being seroprotected at the time of challenge dose. The corresponding percentage observed in the previous studies of this series conducted in 4–5 and 7–8 year-old children were 85.3% and 72.2%, respectively.^4,12^ In a previous study, 78.3% (95% CI: 73.1–83.0) of adolescents who were primed with 3 doses of the HBV vaccine in infancy (0, 1, 6 months schedule) had anti-HBs antibody concentrations ≥10 mIU/mL at the age of 12–13 y.^6^ In the present study, 3 out of 301 children were found to have positive results for anti-HBc antibodies, indicative of a previous HBV exposure; however, it cannot be concluded when the infection occurred.

Despite declining concentrations of circulating anti-HBs antibodies, with increasing age, the vast majority of subjects (96.5%) mounted a strong immune response with a marked increase in GMC following the hepatitis B vaccine challenge. This was comparable to the anamnestic responses mounted by the younger children in the previous studies [4–5 y (96.8%); 7–8 y (96.6%)].^4,12^ Our results are also consistent with the findings of other researchers where strong anamnestic responses against HBV were observed in the majority of individuals many y after priming with either DTPa-HBV-IPV/Hib or monovalent hepatitis B vaccine vaccines.^4,6^^,^^10,12-14^ For example, immune memory following monovalent hepatitis B vaccine administration persisted in approximately 97% subjects in Germany for at least 12–13 y.^6^ In another study conducted in Italy, >96% subjects demonstrated immune memory for 10 y after primary vaccination with monovalent hepatitis B vaccine.^14^

Stratifying anamnestic response according to pre-challenge anti-HBs antibody concentrations, indicated that the anamnestic response mounted by subjects with pre-challenge anti-HBs concentrations <6.2 mIU/mL (92%), 6.2–<10 mIU/mL (100%) and ≥ 10 mIU/mL (98.3%) were comparable to each other. This suggests that pre-challenge antibody concentrations do not have any major effect on the post-challenge antibody concentration. This was further strengthened by the pre- and post-challenge anti-HBs levels correlation analysis where only a moderate correlation between the 2 factors was observed.

A small percentage of subjects (3%) in our study did not mount anamnestic response. However, such low numbers of non-responders have been previously observed.^7,15^

The challenge hepatitis B vaccine dose was well tolerated, and its safety and reactogenicity profile was in line with the known safety profile of monovalent hepatitis B vaccine.^16^

The main limitation of our study is the lack of a control group vaccinated with monovalent hepatitis B vaccine in infancy. Nevertheless, our study supports the available literature where vaccination with of DTPa-HBV-IPV/Hib vaccine in early life can provide long-lasting protection against HBV, despite decreasing antibody levels.

## Material and methods

### Study design

Phase IV, open-label, non-randomized, study conducted between February and September 2014 across 12 centers in Germany (NCT02052661). The study was performed in accordance with Good Clinical Practice Guidelines and the Declaration of Helsinki, and the protocol and study documents were reviewed and approved by local ethics committees. Written informed consent was obtained from parents/guardians before enrolment; the subjects provided written informed assent.

Healthy adolescents aged 12–13 y, previously vaccinated with 4 doses of DTPa-HBV-IPV/Hib vaccine (3 dose primary vaccination by 9 months of age followed by a single booster dose at 11-18 months of age) as part of routine vaccination in Germany, were eligible to participate in the study. In this follow-up study, all enrolled children received a single 0.5 mL challenge dose of monovalent pediatric hepatitis B vaccine (*Engerix*™-B Kinder, GSK Vaccines), containing 10 μg hepatitis B surface antigen (HBsAg), administered intramuscularly in the non-dominant deltoid arm. Children were excluded for the following reasons: participation in another trial; hepatitis B vaccine vaccination since the toddler booster dose; HBV infection; immunosuppressants; immunoglobulins and/or blood products within 3 months before and one month after the study vaccine.

### Immunogenicity assessment

Blood samples were collected immediately before and one month after the challenge dose. Anti-HBs antibody concentrations were determined using a Chemiluminescence immunoassay (CLIA; *Centaur*™, Siemens, Germany) with a 6.2 mIU/mL cut-off; concentrations ≥10 mIU/mL were considered seroprotective. Of note, in vaccinated individuals, anti-HBs levels between 2 and 9.9 mIU/mL may also be considered to be protective.^17^

In addition, anti-HBc antibodies were measured by CLIA on the blood samples collected one month after the challenge.

### Safety and reactogenicity assessment

Subjects recorded solicited local (pain, redness, swelling) and general (fatigue, fever, gastrointestinal symptoms and headache) symptoms for 4 (0–3) days and unsolicited AEs for 31 (0–30) days after the challenge dose in diary cards. The intensity of all AEs was graded on a scale of 1–3; Grade 3 (severe) symptoms were defined as injection site redness or swelling ≥ 50 mm diameter, temperature >39.0 °C and as preventing normal activity for all other symptoms. AEs were recorded during the entire study period.

### Statistical analysis

The immune responses were analyzed for the ATP immunogenicity cohort, which included all eligible subjects who received the challenge dose, complied with protocol-defined procedures and had available post-challenge blood sample results. The analysis for antibody persistence was performed on the ATP persistence cohort, who had available pre-challenge blood sample results.

The reactogenicity and safety analyses were performed on the TVC which included all subjects who received the hepatitis B vaccine challenge dose.

The primary objective of the study was to assess the percentage of subjects who achieved anti-HBs antibody concentrations ≥100 mIU/mL one month post-challenge with exact 95% CIs. The percentage of seropositive and seroprotected subjects, with corresponding GMCs were also calculated. Anamnestic response (defined as a 4-fold or greater increase in the post-challenge anti-HBs antibody concentration in subjects who were seropositive before the challenge dose; or a post-challenge anti-HBs antibody concentration ≥10 mIU/mL in initially seronegative subjects) was also measured one month post-challenge.

The relationship between pre-challenge and post-challenge anti-HBs levels, and between challenge levels i.e., after the booster in the second y of life and post-challenge in this study, was also explored.

Considering a 10% drop out rate (for 300 planned subject), a sample size of 270 evaluable subjects provided 86% power for the lower limit of the 95% CI for >90% subjects to have anti-HBs antibody concentrations ≥100 mIU/mL one month post- challenge, if the true percentage of responders was 95%.

## Trademark

*Infanrix-hexa* and *Engerix-B* Kinder are trademarks of the GSK group of companies.

## Abbreviations

anti-HBs: anti-hepatitis B
ATP: according to protocol
CI: confidence interval
DTPa-HBV-IPV/Hib: diphtheria-tetanus-acellular pertussis-hepatitis B virus-inactivated poliovirus/*Haemophilus influenzae* vaccine
GMC: geometric mean concentration
HBsAg: hepatitis B surface antigen
HBV: hepatitis B virus
WHO: World Health Organization
